# Probing ultra-fast processes with high dynamic range at 4th-generation light sources: Arrival time and intensity binning at unprecedented repetition rates

**DOI:** 10.1063/1.4978042

**Published:** 2017-03-06

**Authors:** S. Kovalev, B. Green, T. Golz, S. Maehrlein, N. Stojanovic, A. S. Fisher, T. Kampfrath, M. Gensch

**Affiliations:** 1 Helmholz Zentrum Dresden Rossendorf, Bautzner Landstr. 400, 01328 Dresden, Germany; 2 Deutsches Elektronen Synchrotron, Notkestr. 85, 22607 Hamburg, Germany; 3 Fritz-Haber-Institut der Max Planck Gesellschaft, Faradayweg 4-6, 14195 Berlin, Germany; 4 SLAC National Accelerator Laboratory, 2575 Sand Hill Rd, Menlo Park, California 94025, USA

## Abstract

Understanding dynamics on ultrafast timescales enables unique and new insights into important processes in the materials and life sciences. In this respect, the fundamental pump-probe approach based on ultra-short photon pulses aims at the creation of stroboscopic movies. Performing such experiments at one of the many recently established accelerator-based 4th-generation light sources such as free-electron lasers or superradiant THz sources allows an enormous widening of the accessible parameter space for the excitation and/or probing light pulses. Compared to table-top devices, critical issues of this type of experiment are fluctuations of the timing between the accelerator and external laser systems and intensity instabilities of the accelerator-based photon sources. Existing solutions have so far been only demonstrated at low repetition rates and/or achieved a limited dynamic range in comparison to table-top experiments, while the 4th generation of accelerator-based light sources is based on superconducting radio-frequency technology, which enables operation at MHz or even GHz repetition rates. In this article, we present the successful demonstration of ultra-fast accelerator-laser pump-probe experiments performed at an unprecedentedly high repetition rate in the few-hundred-kHz regime and with a currently achievable optimal time resolution of 13 fs (rms). Our scheme, based on the pulse-resolved detection of multiple beam parameters relevant for the experiment, allows us to achieve an excellent sensitivity in real-world ultra-fast experiments, as demonstrated for the example of THz-field-driven coherent spin precession.

## INTRODUCTION

Pump-probe experiments employing accelerator-based 4th-generation photon sources and external lasers typically face a temporal jitter of the order of picoseconds (ps) between the accelerator- and laser-generated light pulses. Moreover, the coherent processes underlying the light generation in, e.g., self-amplified spontaneous emission (SASE), superradiant THz emission, or low-gain free-electron lasers (FELs), lead to significant fluctuations of the photon pulse intensities. This has significantly limited the temporal resolution and dynamic range of time-resolved experiments in the past. One approach to deal with this obstacle is to control the accelerator with active feedback loops. Recent pilot experiments lowered the temporal resolutions below 100 fs.^1^ This approach relies on a sophisticated arrangement of fast feedback loops that are challenging to operate routinely. Furthermore, it reaches a physical limit of a few tens of femtoseconds as the timing instability due to mechanical vibrations, microphonics, and temperature drifts in the optical beam transport becomes relevant.

In this article, we pursue a fundamentally different concept based on binning of the relevant pulse parameters and the postmortem or online correction of the taken experimental data. This approach relies on the accurate determination of arrival time and other parameters, such as intensity, for each individual photon pulse. Pulse-resolved detection schemes of this type have been successfully implemented at different XFEL's over the past 15 years.^2–5^ Currently, XFEL facilities can only be operated at comparatively low repetition rates between 120 Hz (Ref. 6) and 8000 Hz.^4^ This on one hand led to comparatively relaxed requirements on the data analysis and data storage speed but on the other hand yields merely a moderate dynamic range as compared to table-top experiments.

Here, we present the first demonstration of binning experimental parameters for every single pulse at the uniquely high repetition rates now available at accelerator-driven photon sources based on superconducting radiofrequency (SRF) technology. We first describe the developed scheme for measuring and subsequent binning of the arrival time and intensity of the accelerator-based photon pulses. Thereafter, we discuss the performance and the arising opportunities based on two benchmark THz pump laser probe experiments: (i) THz time-domain spectroscopy and (ii) transient Faraday probing of a THz driven coherent spin excitation in NiO.

As we show in this article, the instabilities can then in many cases even be turned from a problem into an exciting opportunity for measuring multiple parameter dependencies in parallel. Most importantly, we demonstrate for the first time that ultra-fast accelerator-laser experiments can achieve an excellent dynamic range of up to 120 dB. Our scheme currently works at an unprecedented repetition rate of 100 kHz and with a demonstrated time resolution of better than 13 fs (rms) or 28 fs (FWHM). We achieve this result by a combination of pulse-resolved analysis and homodyne detection. We furthermore demonstrate that the concept of pulse-resolved detection in combination with the high repetition rates available from SRF accelerators allows a highly efficient study of nonlinear transient spectroscopy by determining temporal and fluence dependencies from a single sequence of measurements. Finally, we discuss the current limits of our scheme and show that a temporal resolution in the few-fs regime or better can in principle be achieved.

## PULSE-RESOLVED DETECTION SCHEME

**FIG. 1.**
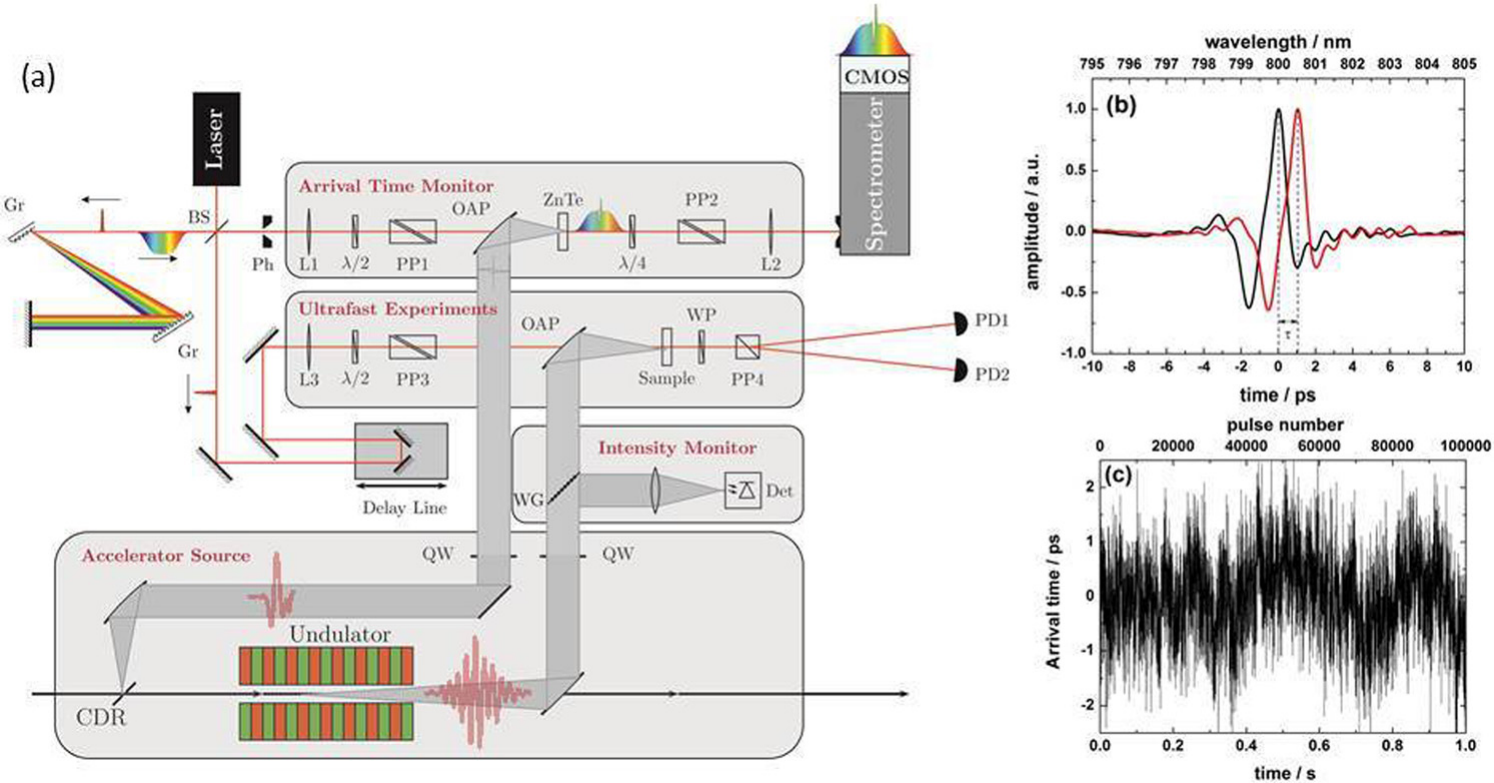
Pulse-to-Pulse detection scheme. (a) Schematic of the experimental setup at TELBE. (b) Spectral-decoding-type electro-optic sampling traces of 2 pulses from the diffraction radiator with an arrival time-difference of τ = 1.03 ps. (c) Pulse-resolved arrival time jitter recorded over 1 s, that is, 10^5^ laser shots.

Figure 1 displays the pulse-to-pulse detection scheme developed for the Terahertz facility at the ELBE accelerator (TELBE).^7^ As we will discuss later in this article, the scheme is readily adjustable to extreme ultraviolet (XUV) and X-ray free electron lasers, but our demonstrator is currently optimized for the THz frequency range. TELBE emits single-cycle pulses by means of a coherent diffraction radiator (CDR) and multicycle THz pulses by means of an undulator, both covering the frequency range between 0.1 and 3 THz at repetition rates as high as 13 MHz. The THz pulses propagate into a dedicated laboratory equipped with various experimental set-ups.^7^ The fs Ti:Sapphire-based amplified laser system is locked to the same master clock as the accelerator,^8^ thereby yielding an intrinsic arrival time jitter and drift of a few ps between the laser pulses and the electron bunches generating the THz pulses (see supplementary material for details). The intensity stability of the TELBE THz sources depends on the accelerator settings. Fluctuations range from <10% (stable operation) to 50% (unstable operation), very similar to the case of SASE X-ray FELs.

In order to perform arrival time measurements at high repetition rates, single-shot electro-optic sampling by means of the spectral decoding technique^9^ is utilized. Compared to other arrival time monitors based on temporal^10,11^ or spatial decoding,^2,3^ spectral decoding places particularly low requirements on the probe-laser pulse energy. It can in principle employ simple oscillator laser systems that are commercially available up to the 100 MHz regime. The primary limitation of spectral decoding in terms of the repetition rate is the read-out rate of the line-array detector measuring the laser-pulse spectrum. We utilize what is to our knowledge the fastest commercially available line-array device, operating up to a rate of 200 kHz.^12^

Our arrival time monitor works as follows: The pulse in one branch of the laser beam is stretched by a grating-based stretcher to a duration of roughly 10 ps and overlapped with the single-cycle THz pulses from the diffraction radiator in a ZnTe crystal where its field is encoded in the spectrum. The spectrum of every second stretched laser pulse is detected by the CMOS line array, and the arrival time is deduced from the position of the main peak (see Figures 1(b) and 1(c)). The pulse duration in the other laser branch laser is kept ultra-short. This pulse is combined with the multi-cycle THz pump pulses from the undulator to probe THz-induced transient dynamics in the sample of choice. In order to perform a pulse-to-pulse background subtraction, we operate the probe laser at 200 kHz, twice the repetition rate of the two accelerator-based THz sources. The intensity of each of the THz pump pulses is determined by a fast pyroelectric detector that can resolve individual THz pulses up to a repetition rate of 200 kHz.^13^

**FIG. 2.**
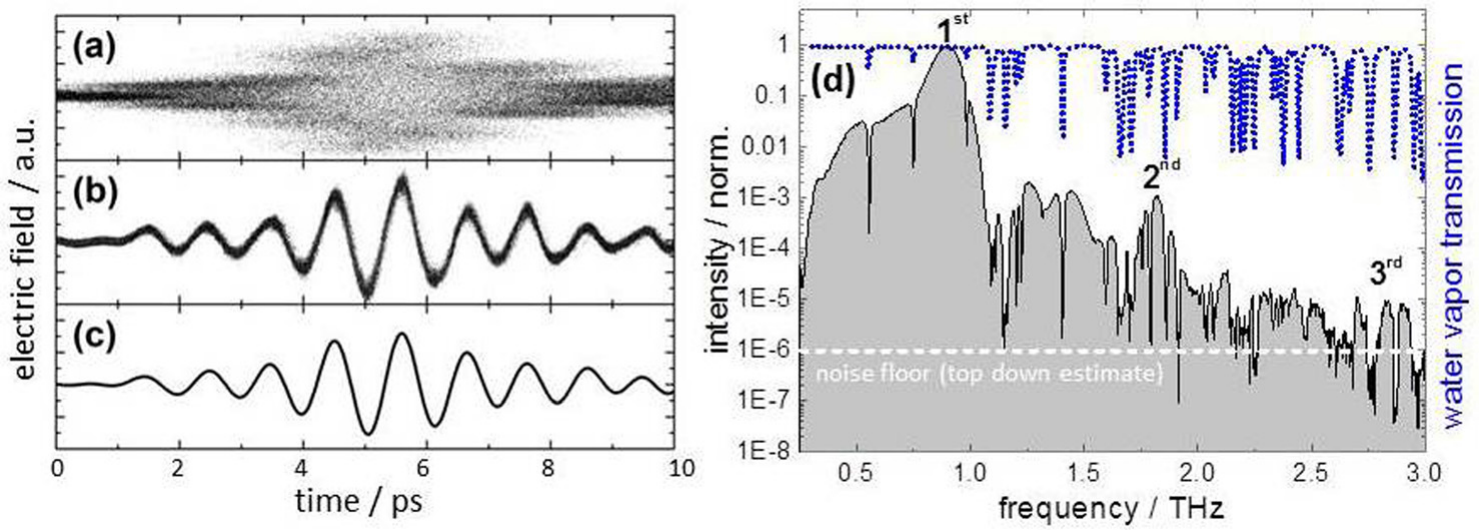
Data-sorting process for the benchmark THz-spectroscopy experiment. The undulator is tuned to a central frequency of 0.9 THz; its electric field is measured by sequential electro-optic sampling in the time domain. (a) Unsorted raw data in which each dot represents a measured value of the THz electric field (y axis) plotted against a time value determined by the optical delay position (x axis). The few-ps jitter prevents us from resolving the individual field cycles. (b) After correcting the time axis for the jitter, the blurring is reduced to below 30 fs (FWHM). The remaining blur is due to the intensity fluctuations during the experiment. (c) Normalized electric-field data obtained by averaging the electric field in bins of 50 fs. (d) Resulting THz frequency-domain spectrum when evaluating the full time-domain measurement over 120 ps, plotted on a logarithmic scale (grey shaded). Higher harmonics up to the 3rd order are observed with a dynamic range of over five orders of magnitude. The measurement was performed in atmosphere, and the narrow water absorption lines (blue dotted line) are clearly resolved up to 3 THz. The narrow dashed white line indicates the top down estimate of the smallest detected signal, which is of the order of 10^−6^. Since the noise floor is clearly lower than this, the maximum dynamic range is 10^6^ or higher.

Figure 2 illustrates the individual steps of the data analysis and benchmarks the achievable sensitivity in a THz time-domain spectroscopy experiment. Time-domain traces of the pulses from the THz undulator tuned to a frequency of 0.9 THz were taken by sequential electro-optic sampling.^14^ The detected time-domain raw data are shown in Figure 2(a). The data resulted from the measurement of few million individual THz pulses, and they are clearly affected and blurred by the arrival time fluctuations between the probing laser and exciting THz pulses. This jitter is on the order of a few ps. After sorting the data by the exact arrival time, only comparatively small field fluctuations in the few-percent regime remain visible as a modest blur of the THz electro-optic signal at each time (see Figure 2(b)). Binning and subsequent normalization of the data lead to the low-noise time-domain signal shown in Figure 2(c) exhibiting a maximum dynamic range of 10^4^ or 80 dB (for a detailed analysis of the achieved dynamic range and signal-to-noise ratio, see supplementary information). Fourier-transformation yields the frequency-domain spectrum shown in Figure 2(d). Note that in this case, the full time-domain measurement over 120 ps was evaluated, thereby leading to a frequency resolution of 8 GHz. The 2nd and 3rd harmonics of the fundamental at 1.8 and 2.7 THz are clearly resolved, and water absorption lines are observed up until 3 THz. The definition of the dynamic range for THz time-domain spectroscopy as given in Ref. 15 is the difference between the maximum signal and noise floor. A top down estimate for the noise floor can be deduced in Figure 2(d) when considering the smallest clearly identifiable THz signal, which in this case is the falling edge of the 3rd harmonic intensity peak; the frequency invariant noise floor clearly must be smaller than this. Thereby, a top down estimate of better than 10^6^ or 120 dB for the maximum dynamic range in the frequency domain can be deduced.

**FIG. 3.**
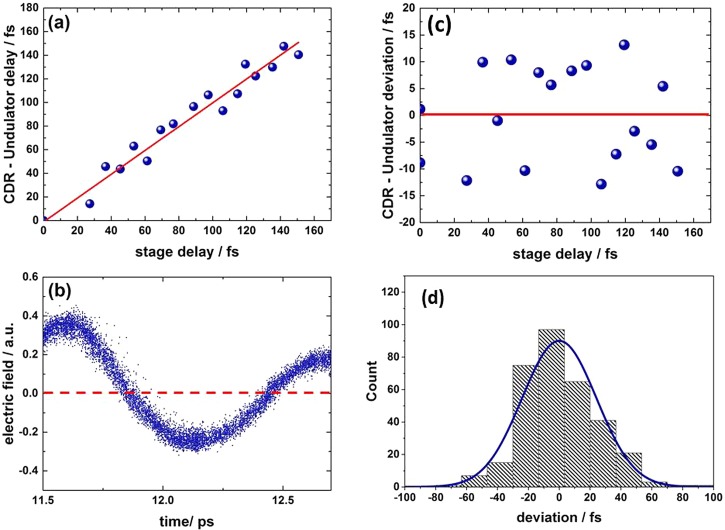
Benchmarking of the time resolution. (a) Measured CDR to undulator radiation delay versus delay position of the ultra-short-pulse beam. The expectation value is given as a red solid line. (b) A zoom into the jitter-corrected electro-optic sampling data of the undulator radiation. (c) Deviation between the expected value from the delay position and the observed time shift between the undulator and CDR. The expectation value is given as a red solid line. (d) Slice through the distribution of the arrival times at the zero-field crossings at 11.8 ps and 12.5 ps (see panel (b)).

The experiment shown in Figure 2 clearly exhibits a time resolution in the sub-ps regime. In the following, we utilize two different methods to establish top-down estimates of the presently achievable time resolution. First, we measured the time delay between the CDR pulses and the pulses from the undulator. This approach makes use of the fact that photon pulses emitted from the same electron bunch can be expected to show an intrinsic synchronization.^16^ The CDR pulse arrival time was then delayed by an optomechanical stage in steps of a few fs, and sequential time-domain measurements of undulator pulses were performed at each step. The observed shift of the multicycle pulses with respect to each other is plotted against the delay stage position in Figure 3(a). The extracted time-shift values vary by 28 fs (FWHM) or 13 fs (rms) from the expectation value (see Figure 3(c)). A second way to estimate the time resolution is to directly measure the linewidth of the jitter-compensated data at zero-field crossings as shown in Figure 3(b). The slice of the distribution with zero intensity shown in Figure 3(d) gives another estimate for the achieved time resolution, which here equals 24 fs (rms) or 56 fs (FWHM), slightly larger than the postulated optimal achievable time resolution, which is due to the contribution of detector noise to the observed electric field values.

An important aspect distinguishing pulse-resolved detection from the more common approach that averages over many pulses is the fact that one can correlate other relevant parameters in addition to the arrival time. This opens up the opportunity to also vary and detect multiple parameters (e.g., pump-pulse intensity, polarization of pump and probe pulses, external magnetic or electric fields, etc.) from one pulse to the next. Accounting for such variation in the data analysis offers the fascinating opportunity of performing simultaneous investigations in a multi-dimensional parameter space. This is particularly fruitful in combination with the high repetition rates provided by existing and future quasi-continuous 4th-generation light sources driven by superconducting radiofrequency (SRF) technology.

**FIG. 4.**
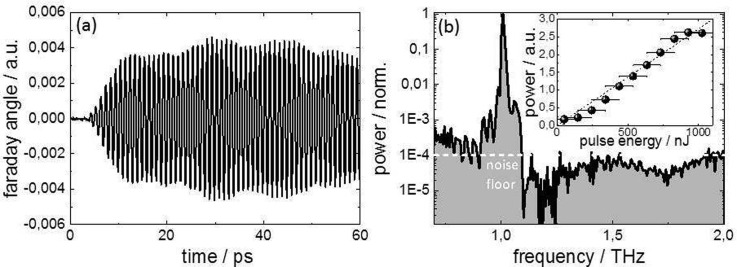
Benchmark THz-pump laser-probe experiment on THz-field-driven coherent spin precession in antiferromagnetic NiO. (a) Transient Faraday measurement of the spin deflection induced by a 1 THz pulse tuned in resonance with the antiferromagnetic resonance. (b) Resulting frequency-domain power spectrum on a logarithmic scale. The white dashed line denotes the noise floor of in the measurement, which according to Ref. 15 allows deducing a maximum dynamic range of 10^4^. The THz pulse energy was varied by between 0 and 1100 nJ during the measurement. By utilizing the pulse-resolved detection of the intensity and the high number (∼10^6^) of pulses involved, one can not only measure the spectrum of the coherent spin wave excitation but simultaneously determine the fluence dependence of the pump-probe signal (see inset).

In Figure 2, we demonstrated a competitive maximum dynamic range of 10^6^ in a time-domain spectroscopy measurement of our THz excitation pulse. These results show that our scheme is suitable for sensitive spectroscopy employing the accelerator-based THz pulses as a probe. A second class of experiments “pumps” a material with a strong THz excitation pulse and “probes” the response with an ultra-short pulse from the laser. In this case, an additional challenge lies in the fact that the detected signal, indicating the pump-induced ultra-fast change, can be minute. The performance of our scheme in this type of experiment has been benchmarked by performing a pump-probe experiment studying coherent spin precession driven by resonant THz excitation. In this experiment, multicycle, narrow-band THz pulses from the undulator source were tuned in resonance with the antiferromagnetic (AFM) mode of the prototypical antiferromagnet NiO at 1 THz. As shown previously,^17^ the THz magnetic field interacts with the electron spins via the Zeeman torque and launches a collective spin excitation at the frequency of the AFM mode.^17^ The net spin deflection can be measured indirectly by the transient Faraday rotation of the polarization plane of probe pulses from an appropriately synchronized femtosecond laser. The results of the measurements utilizing the arrival time and intensity binning at the 100 kHz repetition rate are shown in Figure 4. Linearly polarized laser pulses from the same laser used for the arrival time measurement with a wavelength of 800 nm and a pulse duration of 100 fs were used as an ultrafast probe. The experiment was performed by focusing THz and laser pulses collinearly into a 50 *μ*m thick freestanding NiO crystal. The transient change of polarization is probed by a balanced detection scheme and plotted in Figure 4(a) (for more details on the experiment, see Figure 1(a) and Refs. 7 and 17). The data analysis yields an exceptional dynamic range in the resulting frequency domain spectra of 10^4^ (80 dB), thereby permitting the observation of minute polarization changes induced by the coherently deflected spins (see Figure 4(b)). Evaluating the pulse energy for every pump pulse furthermore allowed the simultaneous determination of the fluence dependence shown as an inset in Figure 4(b). These data are the first example of the advantages of pulse-resolved detection schemes at high repetition rates. The observed linearity is in excellent agreement with the expectations.^17^

## SUMMARY AND OUTLOOK

The scheme and analysis algorithm described in this paper have been employed routinely at the superradiant TELBE THz facility^7^ at repetition rates of up to 100 kHz. Of high general interest is the demonstration of a dynamic range that is competitive to that of table-top laser-based techniques utilizing the conventional phase-sensitive detection. Our results open up the opportunity to measure dependencies of the THz-induced dynamics on multiple parameters at once. The pulse-to-pulse detection scheme presented in this article can be readily transferred to other high-repetition-rate 4th-generation light sources such as quasi-continuous X-ray FELs.^18–20^ The question of whether the arrival time is determined indirectly by spectral encoding of intrinsically synchronized far-field THz pulses as, e.g., emitted by the edge of the X-ray undulators^11^ or by spectral encoding of the direct X-ray arrival time by probing transient electron-hole plasmas excited in solids^5^ or liquids^18^ will depend on the specific boundary conditions (e.g., required time resolution or available space close to the accelerator and access to fs laser pulses).

The main general conclusions of this work are (i) that binning of photon pulse parameters at high repetition rate yields enormous opportunities for vastly improved data quality and (ii) that the substantially higher data rates can be handled in routine user-operation of a large scale photon facility for ultra-fast science. This finding is particularly crucial for the prospect of ultra-fast X-ray science at high-repetition-rate X-ray FELs that are based on quasi-cw SRF technology^19,20^ and are hampered by similar problems with photon pulse parameter instabilities than the TELBE facility. Accordingly, a demonstrator device is currently in development for the European X-FEL.^21^

The technological limit of the temporal resolution in the current scheme of 12 fs (FWHM) or 5 fs (rms) is given by a combination of the probe-pulse bandwidth, the spectrometer, and the pixel size of the CMOS detector. A temporal resolution down to 1 fs or better can readily be achieved as is discussed in detail in the supplementary information. The technological limit of our approach in terms of the repetition rate is currently given by the available CMOS detector arrays. Purpose-built CMOS detector arrays operating at rates up to 900 kHz already exist,^22^ and devices aiming at 4.5 MHz operation are in preparation.^23^

To summarize, we experimentally demonstrate that our pulse-resolved pump-probe detection technique presently provides a temporal resolution of 28 fs (FWHM) or 13 fs (rms) at a repetition rate of 100 kHz, which is close to the current instrumental limit. We achieved a dynamic range in ultra-fast experiments with synchronized external laser systems that is so far unprecedented for accelerator-based photon sources. The underlying pulse-to-pulse detection scheme allows us to perform parallel investigations of dependencies on multiple parameters in one experiment with excellent sensitivity.

## IV. SUPPLEMENTARY MATERIAL

See supplementary material for (i) an analysis of the typical arrival time jitter at TELBE, (ii) a description of how the dynamic range and the SNR are derived in the THz spectroscopy measurement, and (iii) a detailed analysis on where the current limit of the time resolution originates from and how it can be improved in the future.
